# MR-based spatial normalization improves [^18^F]MNI-659 PET regional quantification and detectability of disease effect in the Q175 mouse model of Huntington’s disease

**DOI:** 10.1371/journal.pone.0206613

**Published:** 2018-10-26

**Authors:** Daniele Bertoglio, Jeroen Verhaeghe, Lauren Kosten, David Thomae, Annemie Van der Linden, Sigrid Stroobants, John Wityak, Celia Dominguez, Ladislav Mrzljak, Steven Staelens

**Affiliations:** 1 Molecular Imaging Center Antwerp (MICA), University of Antwerp, Wilrijk, Belgium; 2 Department of Nuclear Medicine, Antwerp University Hospital, Edegem, Belgium; 3 Bio-Imaging Lab, University of Antwerp, Wilrijk, Belgium; 4 CHDI Foundation, Princeton, NJ, United States of America; Centre de Recherche Jean-Pierre Aubert, FRANCE

## Abstract

The positron emission tomography (PET) tracer [^18^F]MNI-659, selective for phosphodiesterase 10A (PDE10A), is a promising tool to assess an early biomarker for Huntington’s disease (HD). In this study we investigated [^18^F]MNI-659 uptake in the Q175 mouse model of HD. Given the focal striatal distribution of PDE10A as well as the striatal atrophy occurring in HD, the spatial normalization approach applied during the processing could sensibly affect the accuracy of the regional quantification. We compared the use of a magnetic resonance images (MRI) template based on individual MRI over a PET and CT templates for regional quantification and spatial normalization of [^18^F]MNI-659 PET images. We performed [^18^F]MNI-659 PET imaging in six months old heterozygous (HET) Q175 mice and wild-type (WT) littermates, followed by X-ray computed tomography (CT) scan. In the same week, individual T_2_-weighted MRI were acquired. Spatial normalization and regional quantification of the PET/CT images was performed on MRI, [^18^F]MNI-659 PET, or CT template and compared to binding potential (BP_ND_) using volumes manually delineated on the individual MR images. Striatal volume was significantly reduced in HET mice (-7.7%, *p*<0.0001) compared to WT littermates. [^18^F]MNI-659 BP_ND_ in striatum of HET animals was significantly reduced (*p*<0.0001) when compared to WT littermates using all three templates. However, BP_ND_ values were significantly higher for HET mice using the PET template compared to the MRI and CT ones (*p*<0.0001), with an overestimation at lower activities. On the other hand, the CT template spatial normalization introduced larger variability reducing the effect size. The PET and CT template-based approaches resulted in a lower accuracy in BP_ND_ quantification with consequent decrease in the detectability of disease effect. This study demonstrates that for [^18^F]MNI-659 brain PET imaging in mice the use of an MRI-based spatial normalization is recommended to achieve accurate quantification and fully exploit the detectability of disease effect.

## Introduction

Huntington’s disease (HD) is an autosomal dominant neurodegenerative disorder characterized by progressive decline in motor function and cognition, and development of psychiatric symptoms [1]. The disease is caused by an expanded CAG repeat in exon 1 of the gene encoding the protein huntingtin (HTT) [2]. Despite the progresses in elucidating the molecular pathology of HD, no disease-modifying therapies are yet available. The main neuropathological feature of HD is the loss of GABAergic medium spiny neurons (MSNs), which represent about 80–90% of striatal neurons [3]. This results in progressive striatal atrophy, followed by cortical degeneration in some patients [4].

Phosphodiesterase 10A (PDE10A) is highly expressed in MSNs, where it regulates intracellular signaling by hydrolyzing the important second messengers cyclic adenosine monophosphate and cyclic guanosine monophosphate [5]. PDE10A has been proposed as an early biomarker and therapeutic target for HD based on the evidence that decreased levels of PDE10A expression occur before the onset of motor-related HD symptoms in transgenic HD mice [6]. In addition, pharmacological inhibition of PDE10A in mouse models of HD improved behavioral and neuropathological abnormalities [7, 8]. The recent development of selective PDE10A radioligands (i.e. [^18^F]MNI-659, (2-(2-(3-(4-(2-[^18^F]fluoroethoxy)phenyl)-7-methyl-4-oxo-3,4-dihydroquinazolin-2-yl)ethyl)-4-isopropoxyisoindoline-1,3-dione) and [^18^F]JNJ42259152) [9, 10] allowed to investigate *in vivo* changes of PDE10A by means of positron emission tomography (PET). HD-related PDE10A decrease at early disease stage has been confirmed by several *in vivo* studies both in mouse models of HD [11–13] and patients with HD [14, 15].

These findings underline the importance of PET imaging in the evaluation PDE10A levels. However, given that PET is an imaging technique of comparatively low spatial resolution and PDE10A expression is mostly limited to the striatum, which is subjected to atrophy in HD, precise image processing and analysis may be challenging to achieve without individual magnetic resonance imaging (MRI). Indeed, co-registration of PET/Computed tomography (CT) with individual MRI, where PET images are fused onto the structural MRI of the same subject, allows taking advantage of the best of both techniques improving signal sensitivity.

Spatial normalization of the PET images and delineation of the volumes of interest (VOIs) are critical steps for group-level statistical analyses. The most accurate method for quantification of PET images requires a dedicated individual MRI for precise co-registration of the PET images and manual delineation of the VOIs. However, this approach is laborious and it could be complicated by inter- and intra-operator variability. Thus, the use of a template for spatial normalization and creation of VOIs is very attractive to standardize the analysis. However, the choice of a specific template warrants caution as various templates are characterized by differences in performance and they might cause the introduction of inaccuracies and under- or overestimations in the quantification.

In this study, we investigated the ability of the PET ligand [^18^F]MNI-659 to detect changes in PDE10A levels at 6 months of age in the recently reported knock-in Q175 animal model for HD [16, 17]. The Q175 mouse model shows motor, cognitive, molecular and electrophysiological abnormalities similar to patients with HD. Given the focal striatal distribution of PDE10A as well as the striatal atrophy occurring in HD, we evaluated different approaches of spatial normalization of the PET data in order to determine which one provides optimal detectability of the disease effect: the first was based on a MRI template generated using the individual MR images, a second on a PET template, and a third using the CT images. The final aim of the study was to validate the most accurate spatial normalization approach for quantification of [^18^F]MNI-659 PET imaging in mice by comparing the binding potential (BP_ND_) values from each method with BP_ND_ quantified in an independent manner using VOIs manually delineated on the individual MR images.

## Materials and methods

### Animals

Heterozygous (HET) six months old male Q175 knock-in mice (*n* = 18; JAX strain name: B6.129S1-Htttm1Mfc/190JChdi) containing the human mutant HTT (mHTT) allele with the expanded CAG repeat within the native mouse Huntington gene [16] and age-matched C57BL/6J wild-type littermates (WT, *n* = 18) were obtained from Jackson Laboratories (Bar Harbour, Maine, USA). The animals were single-housed in individually ventilated cages under a 12 h light/dark cycle in a temperature- and humidity-controlled environment with food and water *ad libitum*. The animals were acclimatized to the facility for at least one week before the start of procedures, which were performed according to the European Committee Guidelines (decree 2010/63/CEE) and the Animal Welfare Act (7 USC 2131). All experiments were approved by the Ethical Committee for Animal Testing (ECD 2014–92) at the University of Antwerp (Belgium).

### T_2_-weighted MRI

To assess atrophy of the striatum and for co-registration purpose, individual MR images were obtained in the same week of the microPET/CT scan. The animals were anaesthetized using isoflurane in a mixture of N_2_/O_2_ (induction 5%, maintenance 1.5%) and placed in prone position onto the scanner (7T Biospec, Bruker, Germany). A rectal thermistor was inserted to monitor the body temperature, which was maintained at 37 ± 1°C by means of a feedback-controlled warm air circuitry (MR-compatible Small Animal Heating System, SA Instruments, Inc. USA). Three-dimensional (3D) turbo rapid acquisition with relaxation enhancement (turboRARE) images were acquired with repetition time 3185 ms, echo time 44 ms, echo train length (ETL) was 8, and matrix size 128 x 64 x 40. Field of view (FOV) was 25.6 x 13 x 10 mm^3^ and resolution of 0.2 x 0.2 x 0.25 mm^3^. Images were acquired using a standard Bruker cross coil set-up with a quadrature volume coil for excitation and an array mouse surface coil for signal detection. The MR image acquisition procedure lasted 21 min. Data were acquired using ParaVision 5.1 (Bruker, Germany).

### [^18^F]MNI-659 microPET imaging

Synthesis of [^18^F]MNI-659 was accomplished by reacting dried [^18^F]Fluoride with the MNI-659 precursor (7 mg) in DMSO (1 ml), followed by purification and formulation into a solution containing propyleneglycol, ethanol, and phosphate buffered saline solution (PBS) as previously described [10]. The specific activity was determined using a UV calibration curve (λ = 230 nm) and was 305 ± 97.66 GBq/μmol.

MicroPET/CT imaging was performed on two Siemens Inveon PET-CT scanners (Siemens Preclinical Solution, USA). An equal number of animals for each genotype was scanned on each PET-CT scanner in a head-to-head position. The animals were anaesthetized using isoflurane (Forene, Belgium) in medical oxygen (induction 5%, maintenance 1.5%). On the day of the scan, body weight was 30.0 ± 1.8 g for WT mice and 28.8 ± 1.4 g for HET Q175 mice (*p* < 0.05). The body temperature of the animals was maintained at 37 ± 1°C during the entire scanning period via a temperature-controlled heating pad. At the onset of the 90 min dynamic microPET scan, cold dose was within tracer conditions (<0.95 μg/kg) with WT mice receiving on average 0.74 ± 0.11 μg/kg and HET mice 0.79 ± 0.16 μg/kg. Thus, the radiotracer was injected intravenously with a bolus of 9.40 ± 4.59 MBq for WT and 6.14 ± 2.52 MBq for HET during 12 s (1 ml/min) using an automated pump (Pump 11 Elite, Harvard Apparatus, USA). Following the microPET scan, a 10 min 80 kV/500 μA CT scan was performed for attenuation correction and for co-registration of the microPET images to the MRI data. The microPET and CT images are co-registered by the scanner software given that the images are acquired on the same microPET/CT scanner. Two WT mice and two HET Q175 animals were excluded from the analysis due to CT-failure. One HET Q175 mouse was not included in the analysis due to faulty tracer injection.

### Image processing and analysis

Acquired PET images were histogrammed and reconstructed into 39 frames of increasing duration: 12x10 s, 3x20 s, 3x30 s, 3x60 s, 3x150 s and 15x300 s. Iterative PET image reconstruction was performed using 4 iterations and 16 subsets of the 2D ordered-subset expectation maximization (OSEM-2D) algorithm [18] following Fourier rebinning. Normalization, dead time, CT-based attenuation and single-scatter simulation scatter corrections were applied. PET image frames were reconstructed on a 128 x 128 x 159 grid with 0.776 x 0.776 x 0.776 mm^3^. For each dynamic scan, a static image was also reconstructed. Images are represented as averages over the group (HET and WT).

PET images were processed and analyzed using PMOD 3.6 software (Pmod Technologies, Zurich, Switzerland). Spatial normalization of the PET/CT images was performed three times independently using a MRI, a PET, and a CT template as described below in order to compare the three approaches.

Using VOIs manually delineated on the MRI template, time activity curves (TACs) of striatum and cerebellum were extracted from the spatially normalized images (S1 Fig). Following kinetic modelling performed with PKIN (PMOD 3.6), the binding potential (BP_ND_) for these regions was calculated using the simplified reference tissue model (SRTM) [19] with the cerebellum as reference tissue.

BP_ND_ values obtained from the MRI, PET, and CT template-based normalized images were subsequently compared to evaluate the impact of spatial normalization on PET uptake quantification. To validate the accuracy of the template-based spatial normalization approaches for noninvasive [^18^F]MNI-659 quantification of BP_ND_ an independent quantification was performed using VOIs that were manually delineated on the individual MR images and calculating BP_ND_. Next, CT images were co-registered to their individual T_2_-weighted MR image through rigid body transformation (rigid matching, mouse changing, interpolation method = trilinear, minimization method = downhill simplex) in PFUS (PMOD 3.6). The transformations were saved and applied to the PET images. Then, TACs were extracted from the individual delineated VOIs (i.e. striatum and cerebellum) using PVIEW (PMOD 3.6). The striatal VOIs were manually delineated on the individual MR images using PVIEW, and they were used to measure changes in striatal volume between genotypes.

As the partial volume effect might affect [^18^F]MNI-659 quantification, BP_ND_ values obtained from the whole striatum from each template-based normalization approach were compared to the values extracted from 50% inner part of the original striatal VOI (focal striatum). In addition, to remove the anatomical boundaries of the striatal VOI, we have analyzed the effect of considering only the hottest 20% of the striatal VOIs on the BP_ND_ quantification. Then, BP_ND_ values were obtained from each template-based normalization approach and compared to the values determined using the VOIs manually delineated on the individual MR images.

#### Creation of [^18^F]MNI-659 PET template

**Fig 1:**
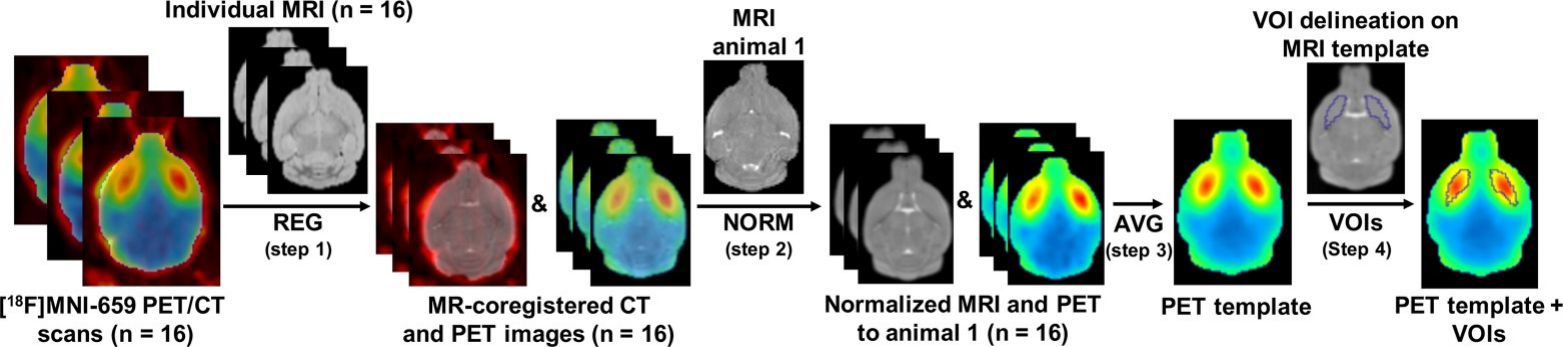
Creation of [^18^F]MNI-659 brain PET template in standardized MR space and VOI delineation. First, static [^18^F]MNI-659 PET images covering the whole scan duration (i.e. 90 min) were generated. Static PET images and the corresponding CT images were co-registered through rigid body transformation to their individual MRI (REG, Step 1) based on CT to MR transformation. Following this step, MRI and PET images were then normalized to the MRI of the first animal through a non-linear warping registration (NORM, Step 2) and were visually inspected for accuracy. Next, the static PET images were averaged in order to obtain the [^18^F]MNI-659 PET template (AVG, Step 3). The averages of all MR images normalized to the first animal were used to generate the MRI template and to delineate manually the VOIs (i.e. striatum and cerebellum). The PET template corresponds and is spatially registered to the MRI template, thus the VOIs defined on the MRI template can be also used in the PET template (Step 4). Unmasked PET images were used for both spatial normalization approaches, however, for visual clarity, masked images are shown. Only data from the WT animals were used. REG = registration, NORM = normalization, AVG = average, WT = wild-type, HET = heterozygous.

In order to investigate the PET template-based spatial normalization, we first created the [^18^F]MNI-659 PET template in standardized MR space with VOIs manually delineated on the MRI template. Only data from the WT animals were used. As Fig 1 shows, static [^18^F]MNI-659 PET scans (*n* = 16) covering the whole scan duration (i.e. 90 min) were generated. CT images were spatially co-registered to individual T_2_-weighted MR images through rigid body transformation (rigid matching, mouse changing, interpolation method = trilinear, minimization method = downhill simplex) in PFUS. This transformation was then applied to the PET images (PET and CT images were intrinsically co-registered as acquired on the same gantry) in order to co-register them to the individual MRI (Step 1). Next, MRI images were normalized to the MRI of the first animal through brain normalization (non-linear warping, 16 iterations, frequency cutoff = 3, regularization = 1) transformation in PFUS and were visually inspected for accuracy (Step 2). The average of all WT MR images normalized to the first animal were used to generate the MRI template on which VOIs (striatum and cerebellum) were manually delineated. Then, both CT and static PET images were normalized to the MRI template using the same MR to MR template transformation as their corresponding individual MR images. The transformed static PET images were averaged in order to obtain the [^18^F]MNI-659 PET template (Step 3). As illustrated in Fig 1, the resulting PET template was spatially registered to the MRI one and the VOIs delineated on the MRI template could also be used in the PET template (Step 4).

#### Template-based spatial normalizations

**Fig 2:**
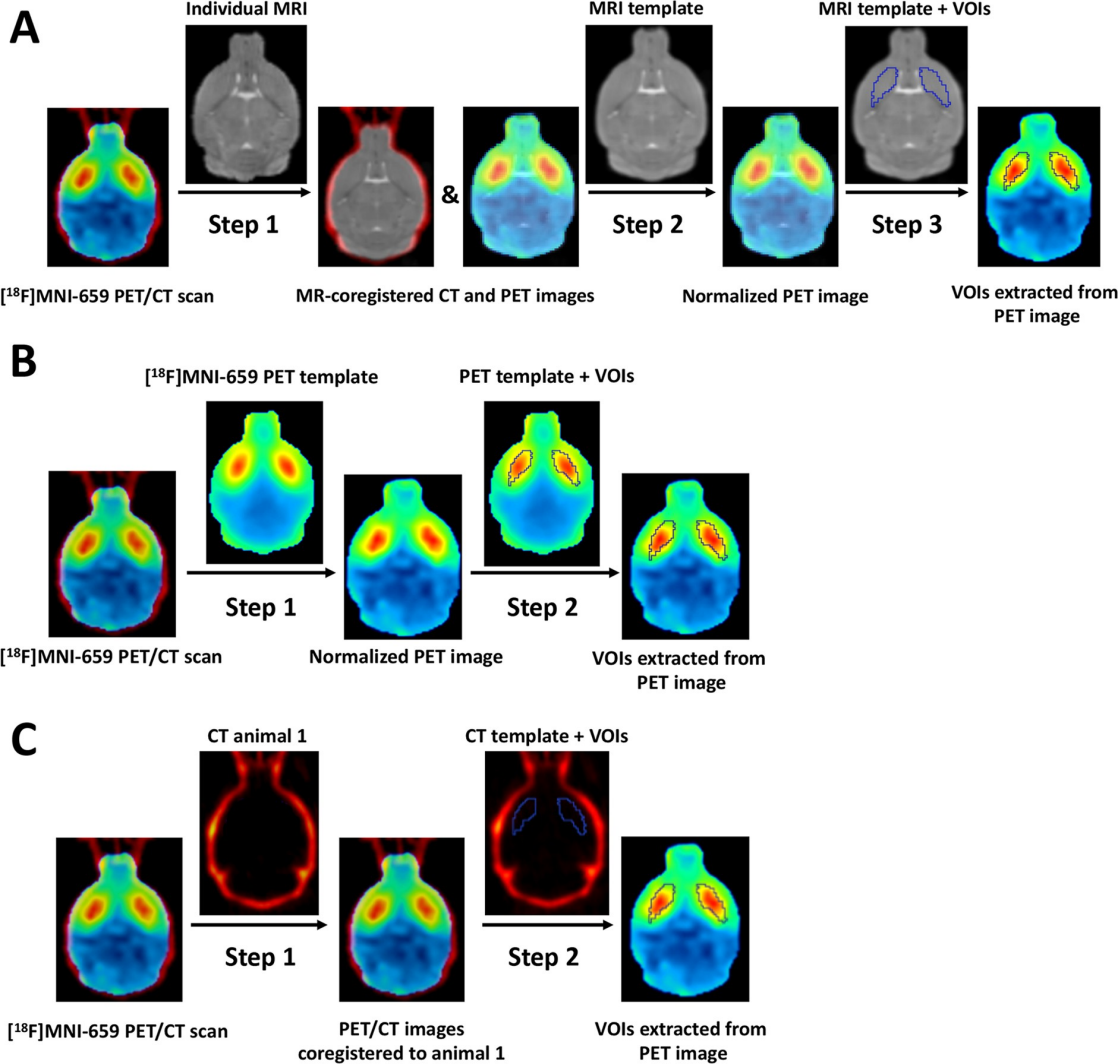
Schematic overview of spatial normalization approaches for [^18^F]MNI-659 regional quantification. (A) MRI template-based spatial normalization: CT images were co-registered to their individual T_2_-weighted MR image through a rigid body transformation and the same transformation was applied to the PET images (Step 1). Then a non-linear warping of the individual MR images to MRI template was performed (Step 2). The same transformation was applied to the [^18^F]MNI-659 PET images in order to obtain the PET images normalized to the MRI templates for extraction of the TACs from the VOIs (Step 3). (B) PET template-based spatial normalization: [^18^F]MNI-659 PET images were normalized through a non-linear warping to the PET template (Step 1) and TACs were extracted from the VOIs (Step 2). (C) CT template-based spatial normalization: CT images were co-registered to the CT of the first animal through a rigid body transformation and the same transformation was applied to the PET images (Step 1). Finally, TACs were extracted from the VOIs (Step 2). Unmasked PET images were used for both spatial normalization approaches, however, for visual clarity, masked images are shown. TACs = time-activity curves.

MRI template-based spatial normalization of the [^18^F]MNI-659 PET images was performed as summarized in Fig 2A. First, using PVIEW brains in PET and CT images were cropped automatically. Next, the CT images were thresholded using PVIEW (replace values <500 with 0) in order to have a clear image of the skull. Then, CT images were co-registered to their individual T_2_-weighted MR image through rigid body transformation (rigid matching, mouse changing, interpolation method = trilinear, minimization method = downhill simplex) in PFUS. The transformations were saved and applied to the PET images (Step 1). Next, brain normalization of the individual MR images to the MRI template was performed in PFUS (non-linear warping, 16 iterations, frequency cutoff = 3, regularization = 1). Transformed images were inspected for accuracy and the transformations were saved. Then, these transformations (i.e. individual MRI to MRI template) were applied to the dynamic [^18^F]MNI-659 PET images (Step 2) in order to obtain the PET images normalized to the MRI template for extraction of the TACs (Step 3).

PET template-based spatial normalization of the [^18^F]MNI-659 PET images was performed as depicted in Fig 2B starting from the same cropped images. Brain normalization of the individual static cropped PET images to the PET template was performed in PFUS (non-linear warping, 16 iterations, frequency cutoff = 3, regularization = 1) (Step 1). Transformed images were inspected for accuracy and the transformations were saved. Finally, transformations were applied to the dynamic images and the TACs were extracted (Step 2).

CT template-based spatial normalization was done using the same cropped images as summarized in Fig 2C. CT images were co-registered to the CT of the first animal through a rigid body transformation (rigid matching, mouse constant, interpolation method = trilinear, minimization method = downhill simplex) and the same transformations were applied to the PET images (Step 1). Next, co-registered PET images were inspected for accuracy and the transformations were saved. Finally, dynamic images were transformed and the TACs were extracted (Step 2). As the MRI template was generated in the space of the first animal, the CT template corresponds and is spatially registered to the MRI template, thus the VOIs defined on the MRI template can be also used for the CT template.

For all spatial normalization approaches WT and HET mice underwent the same processing. Unmasked PET images were used for the registration processes in order to provide as much anatomical information as possible.

### Statistical analysis

All data were assessed for normality (Shapiro-Wilk test). Since no evidence against normality was found, unpaired T-test was used to compare whole brain and striatal volumes between WT and HET Q175 mice. Repeated-measurements ANOVA with Bonferroni correction for multiple comparison was used to investigate regional differences between WT and HET Q175 mice and within each genotype for all spatial normalization approaches. Agreement between BP_ND_ values obtained from the normalization approaches was estimated and visualized by Bland Altman plots as well as Pearson’s correlation tests. In addition, Pearson’s correlation tests were used to examine the correlation between BP_ND_ values based on manually delineated VOIs and the BP_ND_ values derived from the template-based approaches as well as to compare the BP_ND_ values obtained from the whole and 50% or 80% reduced striatal VOIs. Averages and standard errors of the differences as well as 95% confidence intervals (CI) of difference were reported when comparing the normalization approaches. All analyses were performed with GraphPad Prism (v 6.0) statistical software, with the exception of the effect size, which was calculated with G*Power software (http://www.gpower.hhu.de/). The data are represented as mean ± standard deviation (SD) unless specified. All tests were two-tailed and significance was set at *p* < 0.05.

## Results

### Volumetric assessment

**Fig 3:**
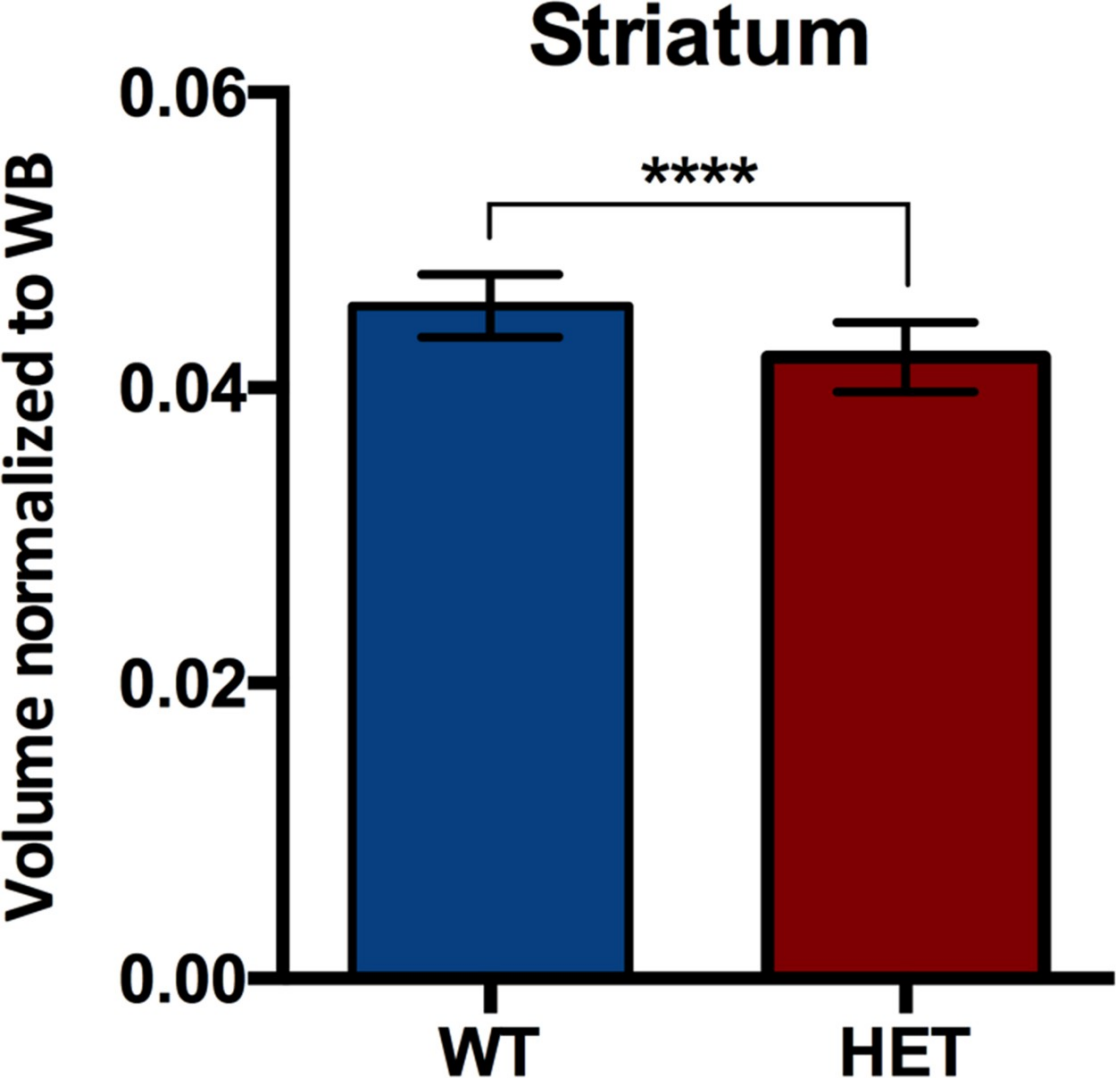
T_2_-weighted MRI displayed reduced striatal volume in 6 months old HET (*n* = 15) compared to WT littermates (*n* = 16). Volumes were manually delineated on the individual MR images. ****p < 0.0001. WT = wild-type, HET = heterozygous, WB = whole brain.

Volumetric assessment of striatum was performed using VOIs manually delineated on the individual MR images and is represented in Fig 3. No difference in whole brain volume was observed between genotypes (*p* = 0.882). Striatal volume normalized to the whole brain was significantly reduced in HET mice compared to WT littermates (*p* < 0.0001), displaying a volume reduction of 7.7% in HET compared to WT mice.

### PET quantification of PDE10A

**Fig 4:**
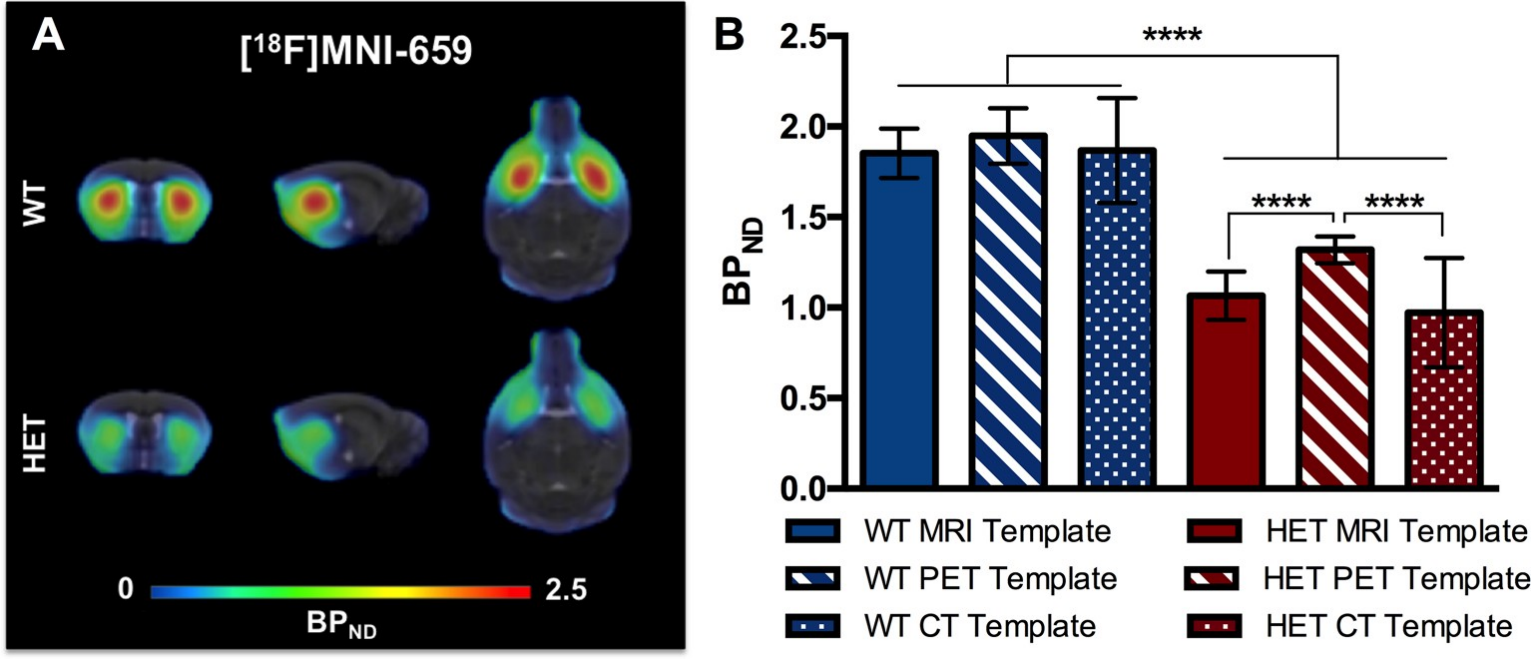
[^18^F]MNI-659 PET quantification. (A) Average [^18^F]MNI-659 BP_ND_ images of 6 months old WT and HET Q175 mice following MRI template-based spatial normalization. PET images are overlaid onto the genotype-specific MRI templates. (B) Striatal [^18^F]MNI-659 BP_ND_ values for HET (*n* = 15) and WT littermates (*n* = 16) Q175 mice of 6 months of age. All spatial normalization approaches (MRI, PET, and CT template-based) showed a significantly reduced [^18^F]MNI-659 binding for HET Q175 mice compared to WT littermates (*p* < 0.0001). In addition, a significant difference between the MRI- and CT-based approaches was found in HET mice when compared to the PET-based approach (*p* < 0.0001). Repeated-measurements ANOVA including Bonferroni correction for multiple comparison. ****p < 0.0001. WT = wild-type, HET = heterozygous.

Average BP_ND_ images of [^18^F]MNI-659 for HET and WT Q175 mice are displayed in Fig 4A. The [^18^F]MNI-659 BP_ND_ values were significantly reduced in HET mice compared to WT animals when using MRI template-based (WT = 1.86 ± 0.20; HET = 1.06 ± 0.24, *p* < 0.0001), PET template-based (WT = 1.93 ± 0.24; HET = 1.32 ± 0.13, *p* < 0.0001), or CT template-based (WT = 1.86 ± 0.29; HET = 0.98 ± 0.32, *p* < 0.0001) spatial normalizations as summarized in Table 1 and shown in Fig 4B.

**Table 1:** Impact of spatial normalization for [^18^F]MNI-659 striatal quantification.

[^18F^]MNI-659 BPND					
	WT	HET	Δ (Genotype)	95% CI of diff	Effect size d
**MRI template**	1.86 ± 0.20	1.06 ± 0.24	0.80 (42.9%)****	+0.533 to +0.962	3.62
**PET template**	1.93 ± 0.24	1.32 ± 0.13	0.61 (31.8%)****	+0.434 to +0.863	3.16
**CT template**	1.86 ± 0.29	0.98 ± 0.32	0.88 (47.3%)****	+0.588 to + 1.030	2.88
**Δ (PET—MRI)**	0.07 (3.7%)	0.25 (23.9%)****			
**Δ (MRI—CT)**	0.00 (0.0%)	0.08 (7.5%)			
**Δ (PET—CT)**	0.07 (3.7%)	0.34 (34.7%)****			

Δ (PET—MRI), difference between MRI and PET template-based spatial normalizations; Δ (MRI—CT), difference between MRI and CT template-based spatial normalizations; Δ (PET—CT), difference between PET and CT template-based spatial normalizations; Δ (Genotype), difference between HET and WT Q175 mice; CI of diff, confidence intervals of difference; WT, wild-type; HET, heterozygous.

****p < 0.0001.

MRI template-based spatial normalization resulted in an average decrease of 42.9 ± 10% (95% CI of difference = +0.533 to +0.962) in the striatum of HET mice compared to WT littermates at 6 months of age. PET template-based spatial normalization showed a decrease in the striatum of HET mice (average decrease of 31.8 ± 13.1%; 95% CI of difference = +0.434 to +0.863) compared to WT littermates at 6 months of age. Finally, CT template-based spatial normalization resulted in an average decrease of 47.3 ± 11.8% (95% CI of difference = +0.588 to +1.030) in the striatum of HET mice compared to WT littermates at 6 months of age.

The MRI-based approach showed the largest effect size (d = 3.62) given the large average difference and limited standard deviation of the groups. The PET-based approach resulted in a lower effect size (d = 3.12) due to the reduced average difference between genotypes. Finally, the CT-based approach showed the lowest effect size (d = 2.88) because of the larger standard deviation in each investigated group (Table 1).

### Impact of spatial normalization for PET quantification

**Fig 5:**
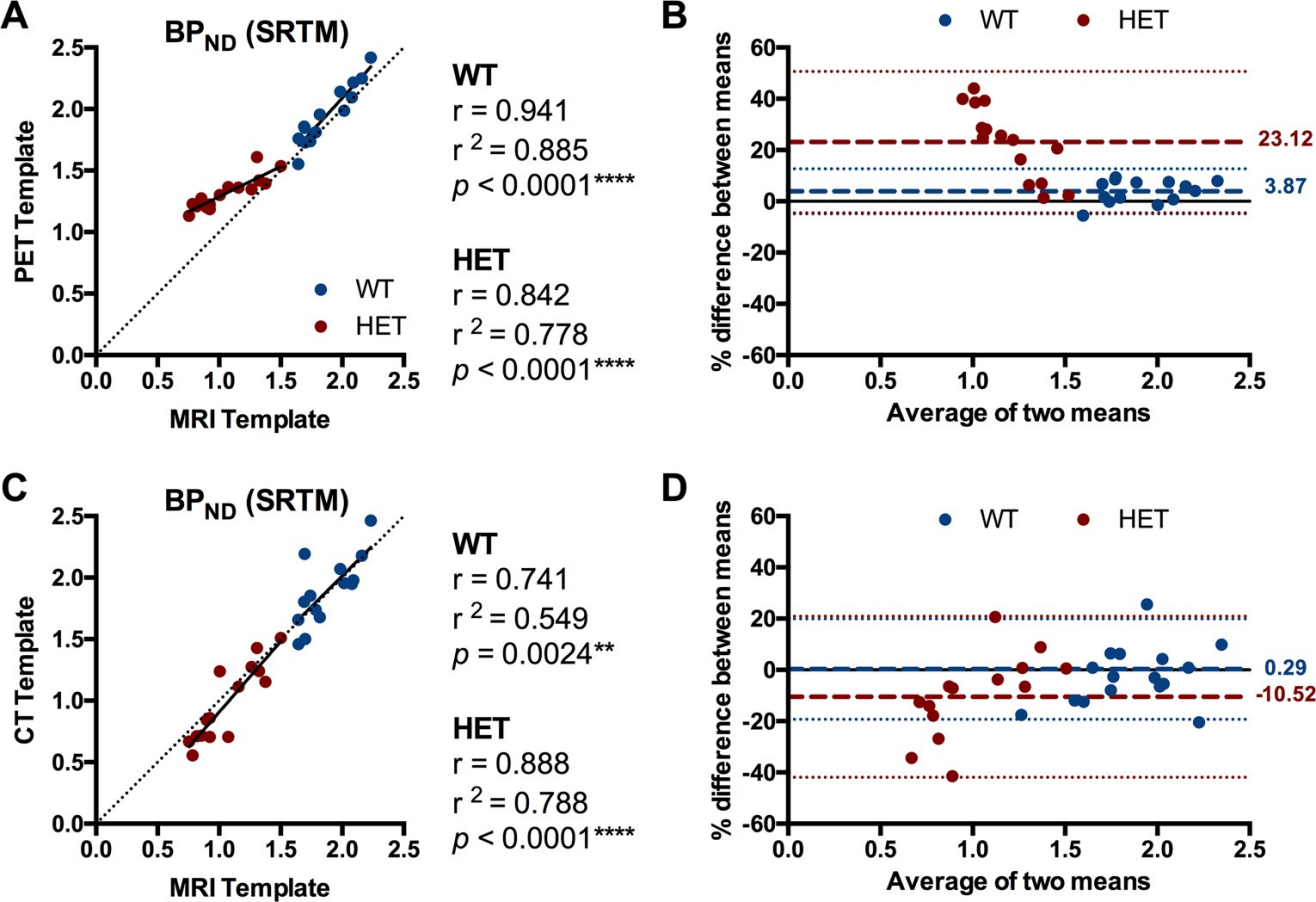
Impact of spatial normalization on [^18^F]MNI-659 BP_ND_. (A) Correlation between striatal [^18^F]MNI-659 BP_ND_ values obtained from MRI- and PET-based spatial normalizations for WT and HET Q175 mice. The regression line of HET Q175 mice sensibly deviated from the identity line (dotted line) at lower values. (B) Bland Altman plot to compare the MRI- and PET-based approaches of spatial normalizations for [^18^F]MNI-659. HET Q175 mice were characterized by a relevant deviation between the two approaches, while WT littermates showed high agreement between measurements. (C) Correlation between striatal [^18^F]MNI-659 BP_ND_ values obtained from MRI- and CT-based spatial normalizations for WT and HET Q175 mice. (D) Bland Altman plot to compare the MRI- and CT-based approaches of spatial normalizations for [^18^F]MNI-659. HET Q175 mice were characterized by a deviation between the two approaches, while WT littermates showed agreement between measurements. Dotted lines represent the 95% limits of agreement (mean difference ± 1.96 x SD of the differences). The bias between the two approaches is represented by the gap between the mean (red and blue dashed lines for HET and WT, respectively) and X axis (solid line). The solid horizontal line indicates y = 0. WT = wild-type, HET = heterozygous.

The results of the different spatial normalizations for [^18^F]MNI-659 are summarized in Table 1 and Fig 4B. The BP_ND_ values in striatum were higher in both WT and HET when analyzed using the PET template-based spatial normalization. This normalization-based difference was pronounced and statistically significant in the HET Q175 mice when compared to both the MRI template (*p* < 0.0001; +23.9 ± 3.2%; 95% CI of difference = +0.043 to +0.465) as well as the CT template (*p* < 0.0001; +34.7 ± 2.6%; 95% CI of difference = +0.080 to +0.615) (Table 1). WT mice displayed only a negligible normalization-based change when compared to the MRI-based (*p* = 0.37; +3.7%; 95% CI of difference = -0.211 to +0.048) or CT-based (*p* = 0.70; +3.7%; 95% CI of difference = -0.067 to +0.192) quantification (Table 1). As a result, the BP_ND_ difference between HET and WT Q175 mice was reduced when using the PET template-based normalization instead of the MRI template-based one. In addition, the CT template-based approach was characterized by an increased standard deviation for both WT and HET animals, thus requiring larger group sizes to obtain the same statistical power. Overall BP_ND_ values obtained using the PET and MRI template-based normalization strategies significantly correlated (WT: r = 0.941, r^2^ = 0.885, and *p* < 0.0001; HET: r = 0842, r^2^ = 0.778, and *p* < 0.0001), however the regression line sensibly deviated from the identity line for HET Q175 mice (Fig 5A), especially towards the lower activities. The deviation between these two normalization strategies of the HET Q175 mice can also be appreciated with a Bland Altman plot, where the bias between the two approaches is represented by the gap between the mean (red and blue dashed lines for HET and WT, respectively) and the X axis (23.12% and 3.87% for HET and WT, respectively) (Fig 5B). On the other hand, BP_ND_ values obtained using the CT template-based normalization approach significantly correlated with the MRI template-based ones (WT: r = 0.741, r^2^ = 0.549, and *p* = 0.0024; HET: r = 0.888, r^2^ = 0.788, and *p* < 0.0001), however the correlations were only moderate due to a more scattered distribution (Fig 5C). The Bland Altman plot based on the CT and MRI template-based approaches underlined this variability as visible by the large 95% confidence intervals (dotted lines, Fig 5D).

### Accuracy of the normalization approaches for [^18^F]MNI-659 quantification

**Fig 6:**
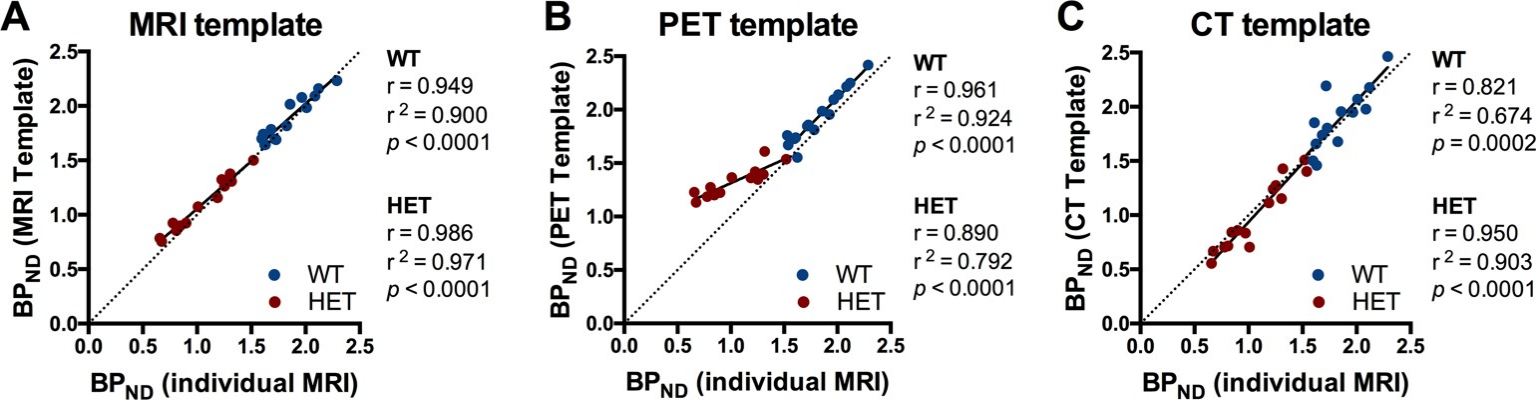
Accuracy of normalization approaches to quantify [^18^F]MNI-659. BP_ND_ of [^18^F]MNI-659 using striatal VOI manually delineated on the individual MR images were calculated to investigate accuracy of the spatial normalization approaches. BP_ND_ values showed strong significant correlations with the MRI template-based approach for both WT and HET mice (r = 0.949 and r = 0.986, respectively) (A) as well as with the PET template-based approach for WT mice (r = 0.961), while HET mice (r = 0.890) sensibly deviated from the identity line (B). Finally, significant correlations were found when using the CT-based approach for both WT and HET mice (r = 0.821 and r = 0.950, respectively) (C). Pearson’s correlation tests. Dotted line represents identity line. WT = wild-type, HET = heterozygous.

The BP_ND_ values determined using the MRI template-based approach showed strong significant correlations with BP_ND_ based on individual MRI (WT: r = 0.949, r^2^ = 0.900, *p* < 0.0001; HET: r = 0986, r^2^ = 0.971, *p* < 0.0001) (Fig 6A). Similarly, BP_ND_ values of WT mice determined using the PET template-based approach showed robust correlation (r = 0.961, r^2^ = 0.924, *p* < 0.0001); however, HET mice showed less significant correlations with BP_ND_ based on individual MRI (r = 0.890, r^2^ = 0.792, *p* < 0.0001) with clear deviation from the identity line (Fig 6B). Finally, the BP_ND_ values calculated using the CT template-based approach showed less significant correlations with BP_ND_ based on individual MRI for WT mice (r = 0.821, r^2^ = 0.674, *p* < 0.0001), but not HET animals (r = 0.950, r^2^ = 0.903, *p* < 0.0001) (Fig 6C).

Additionally, to exclude that partial volume effect might affect one or another approach, BP_ND_ values for the whole striatum and a focal striatum were compared. Strong significant correlations were found between [^18^F]MNI-659 BP_ND_ values based on the whole striatum and the focal striatum when considering the different approaches: MRI template (r = 0.986, *p* < 0.0001 and r = 0.978, *p* < 0.0001 for WT and HET mice, respectively) (Panel A in S2 Fig), PET template (r = 0.971, *p* < 0.0001 and r = 0.961, *p* < 0.0001 for WT and HET mice, respectively) (Panel B in S2 Fig), and CT template (r = 0.982, *p* < 0.0001 and r = 0.990, *p* < 0.0001 for WT and HET mice, respectively) (Panel C in S2 Fig), indicating that the VOI size did not change the outcome. In addition, there was no correlation between the difference of BP_ND_ values obtained using the different spatial normalization approaches and the striatal volumes delineated (MRI- vs PET-based: r = 0.284, r^2^ = 0.08, *p* = 0.286; MRI- vs CT-based: r = 0.100, r^2^ = 0.001, *p* = 0.728).

Finally, to remove the anatomical boundaries of the striatal VOI, we have analyzed the effect of considering only the hottest 20% of the striatal VOIs on the BP_ND_ quantification (S3 Fig). The resulting correlations were comparable to the ones obtained considering the whole striatal VOI (Fig 6), with an expected increase of the values due to the smaller VOI: MRI template (r = 0.915, *p* < 0.0001 and r = 0.881, *p* = 0.0002 for WT and HET mice, respectively) (Panel A in S3 Fig), PET template (r = 0.830, *p* = 0.0002 and r = 0.434, *p* = 0.1388 for WT and HET mice, respectively) (Panel B in S3 Fig), and CT template (r = 0.795, *p* = 0.0007 and r = 0.895, *p* < 0.0001 for WT and HET mice, respectively) (Panel C in S3 Fig). These results suggest that the VOI delineation and size did not change the outcome.

## Discussion

To date, no studies to directly validate the influence of MRI for [^18^F]MNI-659 PET quantification have been performed. In the present work, we prospectively evaluated the BP_ND_ values after MRI, PET, and CT template-based spatial normalization of HET mice and WT littermates. We found that the PET template-based spatial normalization resulted in significantly higher BP_ND_ values in striatum of HET Q175 mice. The CT template-based approach did not affect the group-average quantification, however ensued in a larger variability at the group levels.

Since dedicated high resolution small animal MR scanners are not often available in proximity of preclinical PET centers, the use of a ligand-specific PET template is frequently the preferred choice and it has proved to provide high accuracy results [20–24]. However, likely due to the focal uptake of [^18^F]MNI-659 and the substantial signal reduction in HET Q175 mice, the performance of the PET template is not sufficient to ensure proper quantification. A valid alternative could be the use of the CT images for spatial normalization since they are acquired in parallel with the PET images for attenuation correction [25]. Although this approach might be easy to apply, it lacks detailed information of the brain due to the limited contrast of the brain tissue. Indeed, given the high intensity of the signal in the skull, this is the only structure to drive the CT-based spatial normalization [26]. As a consequence of the lack of spatial information within the brain structures, the performance of this approach can provide an overall reliable group-level quantification, but it is not accurate when looking at the specific subject.

A major hallmark of HD is loss of projection neurons in the striatum, with consequent striatal atrophy [4]. Given that PDE10A is expressed in MSNs in the striatum, structural changes could potentially affect the PET quantification. In the present study, we found decreased striatal volume of HET Q175 mice as previously reported in animal models of HD [16, 27, 28]. A cross-sectional study in patients with early HD demonstrated a relationship between striatal [^18^F]MNI-659 uptake and regional brain atrophy (r = 0.667, *p* < 0.05) [15]. However, this was not the case in present study.

In this study, we found a statistically significant decrease in [^18^F]MNI-659 BP_ND_ values in HET mice compared to WT littermates at 6 months of age using all spatial normalization approaches (-42.8%, -31.8%, and -47.3% for MRI, PET, and CT template-based, respectively). This is in line with the recent literature where a decrease of 40 to 50% in binding of PDE10A in the striatum has been reported in animal models of HD [11–13] as well as in patients with HD [14, 15, 29]. When we evaluated the differences in BP_ND_ values between the spatial normalization approaches, we found that PET template-based normalization resulted in statistically significant higher BP_ND_ values in striatum of the HET mice compared to the other two approaches (*p* < 0.0001). This deviation in HET Q175 mice was clearly visible when using the Bland Altman plot.

Nonetheless, a comparison of the approaches to an independent measurement is required to ensure which method is more accurate in obtaining the striatal binding potential. To this end, we quantified BP_ND_ with VOIs manually delineated on the individual MR images since the values obtained with this approach should represent the reference methods for BP_ND_ quantification of [^18^F]MNI-659. Interestingly, the MRI template-based approach was almost in perfect agreement with BP_ND_ (r > 0.94 for both genotypes). The PET template-based approach resulted in weaker correlations with BP_ND_ for HET Q175 mice (r = 0.890) with a clear deviation from the identity line, and the CT template-based approach showed moderate correlation for WT mice (r = 0.821).

These evidences suggest that PET template-based normalization introduces some deviations at lower activities, possibly due to the very low spatial information in the PET images, resulting in a less accurate normalization. Although this observed overestimation of the BP_ND_ values at lower activities may seem counterintuitive, it is likely to be linked to the mismatch between the PET template and the individual HET PET images. The software may introduce changes during the spatial normalization in order to compensate for the decreased signal, causing a deformation of the image in order to better fit it to the PET template. Alternatively, it might enhance the spillover from outside into the striatum to increase the activity and better match the template. Since at very low radiotracer uptakes there was a larger mismatch, this effect might be amplified with the reduction of the uptake. Consequently, the PET template-based approach is characterized by an overestimation of the striatal binding potential at lower activities, which translates in a decline of the capacity to detect the disease effect.

Unlike the PET template approach, the CT-based spatial normalization did not introduce deviations from the identity line, however the BP_ND_ values showed a larger variability. Thus, the CT template-based approach resulted in a reduced statistical power, thus lowering the detectability of the disease effect.

[^18^F]MNI-659 PET imaging is a promising noninvasive tool to detect early HD. It may be employed to monitor longitudinal changes and as treatment read-out when testing efficacy of novel HD therapies. For these reasons, it is important to apply the most accurate spatial normalization approach in order to avoid the introduction of biases that could lead to the misinterpretation of results. For instance, the overestimation of BP_ND_ values introduced by the PET template-based spatial normalization fails to accurately quantify the striatal BP_ND_ at lower values. Consequently, the temporal decline during a longitudinal study or the efficacy of a novel therapy in preventing PDE10A decline could be underestimated.

PDE10 has been detected as one of the earliest and most profoundly downregulated gene in mouse models of HD [6, 17, 30–32]. This reduction in PDE10A levels is not only related to neuronal loss in the striatum, but it has been suggested to be related to interference of mHTT with the transcriptional machinery of PDE10A, leading to an altered pattern of gene expression followed by neuron dysfunction and death [33]. In addition, [^18^F]MNI-659 binding strongly correlates with markers of disease severity [15]. Interestingly, a recent longitudinal study in patients with HD showed an average 15% decline in [^18^F]MNI-659 binding potential in the caudate suggesting caudate as a sensitive marker of early premanifest pathology or prediction of the motor manifestation [29]. Finally, [^18^F]MNI-659 is characterized by excellent brain penetration, good specificity for PDE10A, a high signal to background ratio, and test-retest reliability [10, 15]. Given the potential application of this PET tracer to monitor an early biomarker for HD, and considering its possible application to predict treatment response, it is fundamental to exploit its potential by determining the optimal spatial normalization to detect disease effect.

In conclusion, this study demonstrates that for [^18^F]MNI-659 brain PET imaging in mice the use of a PET or CT template-based approach results in a lower accuracy of BP_ND_ quantification with overestimation of binding potential when tracer uptake is significantly reduced or increased variability with reduced statistical power, respectively. Thus, the use of an MRI-based spatial normalization is recommended to achieve accurate quantification and higher detectability of disease effect.

## Supporting information

S1 Fig [^18^F]MNI-659 SUV TACs. Average SUV TACs for striatum (full lines) and cerebellum (dotted lines) of WT (*n* = 16) and HET (*n* = 15) Q175 mice following MRI, PET and CT template-based approaches. Data are represented as mean ± standard error mean. WT = wild-type, HET = heterozygous.
(TIFF)

S2 Fig Effect of striatal volume delineation on [^18^F]MNI-659 quantification. Correlation between [^18^F]MNI-659 BP_ND_ values based on the whole striatum and the 50% volume reduced inner part (focal striatum) showed strong significant correlations when considering all approaches indicating that the VOI size did not affect the outcome. (A) MRI template (r = 0.986, *p* < 0.0001 and r = 0.978, *p* < 0.0001 for WT and HET mice, respectively), (B) PET template (r = 0.971, *p* < 0.0001 and r = 0.961, *p* < 0.0001 for WT and HET mice, respectively), and (C) CT template (r = 0.982, *p* < 0.0001 and r = 0.990, *p* < 0.0001 for WT and HET mice, respectively). Dotted line represents identity line. WT = wild-type, HET = heterozygous.
(TIFF)

S3 Fig Effect of striatal volume on [^18^F]MNI-659 quantification. BP_ND_ of [^18^F]MNI-659 using striatal VOI manually delineated on the individual MR images were compared to the hottest 20% of the striatal VOIs for each spatial normalization approach. BP_ND_ values showed strong significant correlations with the MRI template-based approach for both WT and HET mice (r = 0.915 and r = 0.881, respectively) (A) as well as with the PET template-based approach for WT mice (r = 0.830), while HET mice did not (r = 0.434) and they sensibly deviated from the identity line (B). Finally, significant correlations were found when using the CT-based approach for both WT and HET mice (r = 0.795 and r = 0.895, respectively) (C). Pearson’s correlation tests. Dotted line represents identity line. WT = wild-type, HET = heterozygous.
(TIFF)
