# Supplementary figures and images for: MR-based spatial normalization improves [18F]MNI-659 PET regional quantification and detectability of disease effect in the Q175 mouse model of Huntington’s disease
Source: PLoS One. 2018 Oct 26;13(10):e0206613. doi: 10.1371/journal.pone.0206613 (PMC6203386; doi:10.1371/journal.pone.0206613)

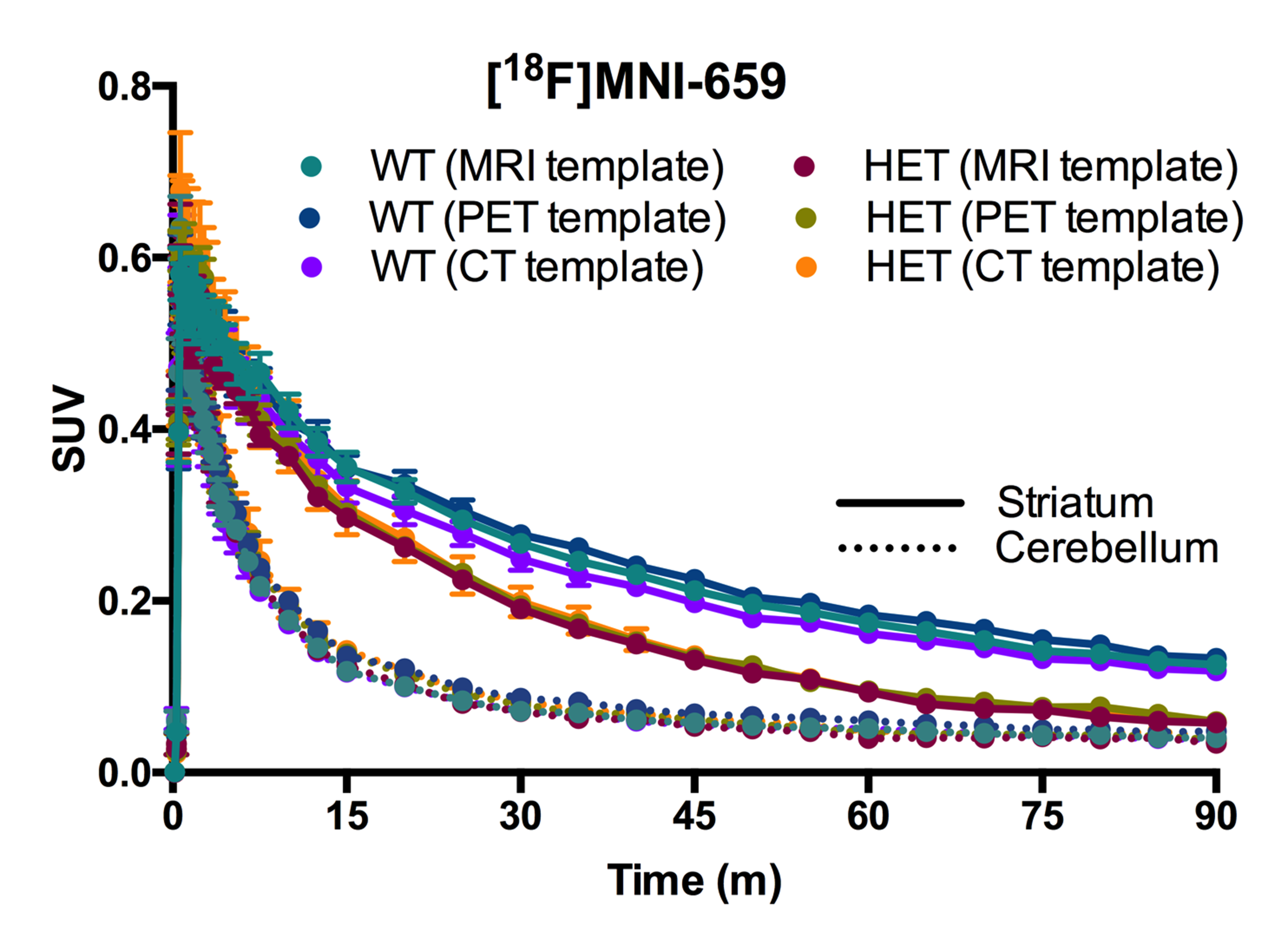

Supplement: S1 Fig — Average SUV TACs for striatum (full lines) and cerebellum (dotted lines) of WT (n = 16) and HET (n = 15) Q175 mice following MRI, PET and CT template-based approaches. Data are represented as mean ± standard error mean. WT = wild-type, HET = heterozygous. (TIFF) [file pone.0206613.s001.tiff]

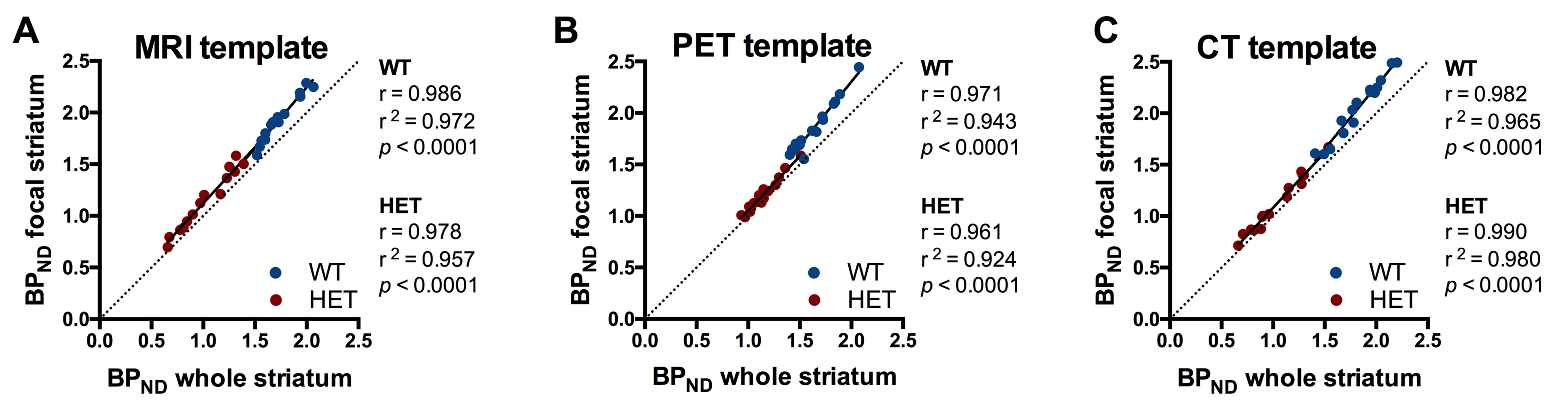

Supplement: S2 Fig — Correlation between [18F]MNI-659 BPND values based on the whole striatum and the 50% volume reduced inner part (focal striatum) showed strong significant correlations when considering all approaches indicating that the VOI size did not affect the outcome. (A) MRI template (r = 0.986, p < 0.0001 and r = 0.978, p < 0.0001 for WT and HET mice, respectively), (B) PET template (r = 0.971, p < 0.0001 and r = 0.961, p < 0.0001 for WT and HET mice, respectively), and (C) CT template (r = 0.982, p < 0.0001 and r = 0.990, p < 0.0001 for WT and HET mice, respectively). Dotted line represents identity line. WT = wild-type, HET = heterozygous. (TIFF) [file pone.0206613.s002.tiff]

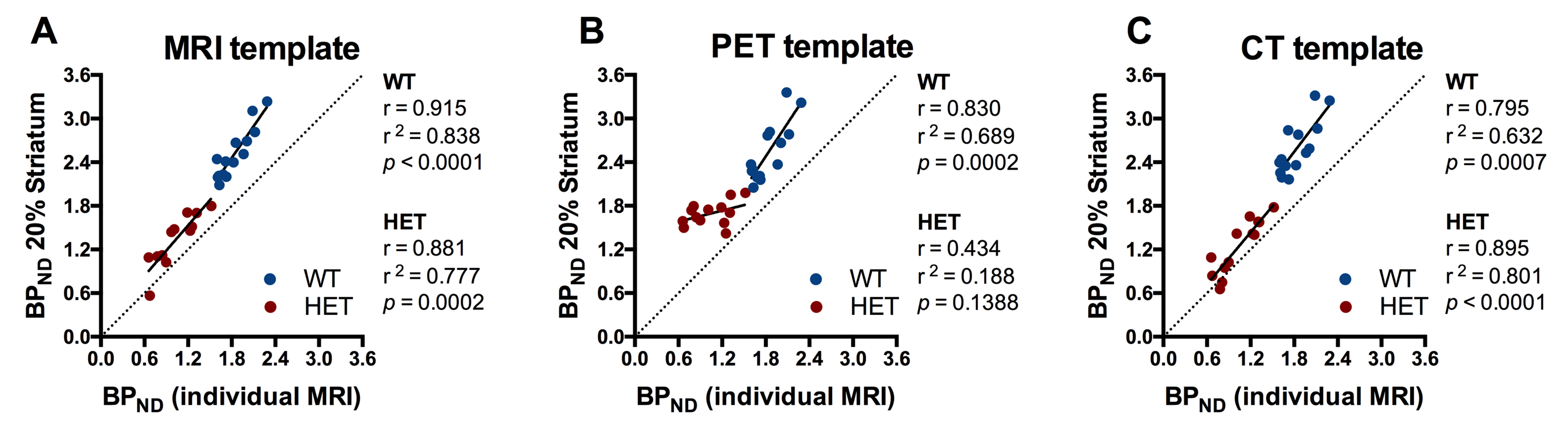

Supplement: S3 Fig — BPND of [18F]MNI-659 using striatal VOI manually delineated on the individual MR images were compared to the hottest 20% of the striatal VOIs for each spatial normalization approach. BPND values showed strong significant correlations with the MRI template-based approach for both WT and HET mice (r = 0.915 and r = 0.881, respectively) (A) as well as with the PET template-based approach for WT mice (r = 0.830), while HET mice did not (r = 0.434) and they sensibly deviated from the identity line (B). Finally, significant correlations were found when using the CT-based approach for both WT and HET mice (r = 0.795 and r = 0.895, respectively) (C). Pearson’s correlation tests. Dotted line represents identity line. WT = wild-type, HET = heterozygous. (TIFF) [file pone.0206613.s003.tiff]
